# Normothermic regional perfusion for donation after circulatory death in lung transplantation

**DOI:** 10.3389/fcvm.2025.1716890

**Published:** 2025-11-28

**Authors:** Andrew J. Gorton, Daniel K. Mohammadi, Mohammad J. Malik, Suresh Keshavamurthy

**Affiliations:** 1 Division of Cardiothoracic Surgery, Department of Surgery, University of Kentucky, Lexington, KY, United States; 2 Department of Cardiovascular and Thoracic Surgery, The University of Texas Southwestern Medical Center, Dallas, TX, United States

**Keywords:** lung tranplant, normothermic regional perfusion (NRP), extracorporeal mebrane oxygenation, donation after circulatory death (DCD), tranplant

## Abstract

Donor supply of lung allografts lags behind the waitlist demand leading to an ongoing attempt to expand the donor pool. Recently this has mean increased utilization of donation after circulatory death (DCD). The concern about graft quality with this approach is more acute in fields with organs especially sensitive to ischemia, such as the lungs. One strategy being utilized to limit ischemic time is *in situ* thoracoabdominal normothermic regional perfusion with the use of extracorporeal membrane oxygenation (ECMO) or cardiopulmonary bypass (CPB) instituted after declaration of circulatory death to restore organ perfusion. This method is thought to decrease ischemic time, allow for correction of metabolic abnormalities, and provide longer for organ procurement. The data evaluating both the graft function and clinical outcomes following donation after circulatory death with normothermic regional perfusion (DCD-NRP) are growing with early results suggesting equivalent graft recovery and similar survival. This review aims to gather details of the procedure utilization, graft function, and patient outcomes and summarize the outcomes from a growing pool of data.

## Introduction

The number of people on the lung transplant wait list continues to grow at a higher rate than the number of lung transplants performed annually. Despite recent changes in the lung allocation score and increased utilization of donation after circulatory death (DCD), a sizeable void persists between the total number of candidates and actual lung transplant recipients. Further compounding the issue, roughly 15% of all lung transplants in 2024 were from DCD donors, leaving a significant number of DCD donor lungs that were not transplanted (1, 2). One of the main factors leading to organ rejection is organ quality. One strategy being utilized to limit ischemic time is *in situ* thoracoabdominal normothermic regional perfusion with the use of extracorporeal membrane oxygenation (ECMO) or cardiopulmonary bypass (CPB) instituted after DCD. This method is thought to decrease ischemic time, allow for correction of metabolic abnormalities, and provide longer for organ procurement (1, 2). Although extensively studied with other solid organ transplants, like the kidney and liver, there does not exist such abundance of evidence for lung transplant outcomes following DCD donors procured with normothermic regional perfusion (NRP) support (2–4).

**Figure 1:**
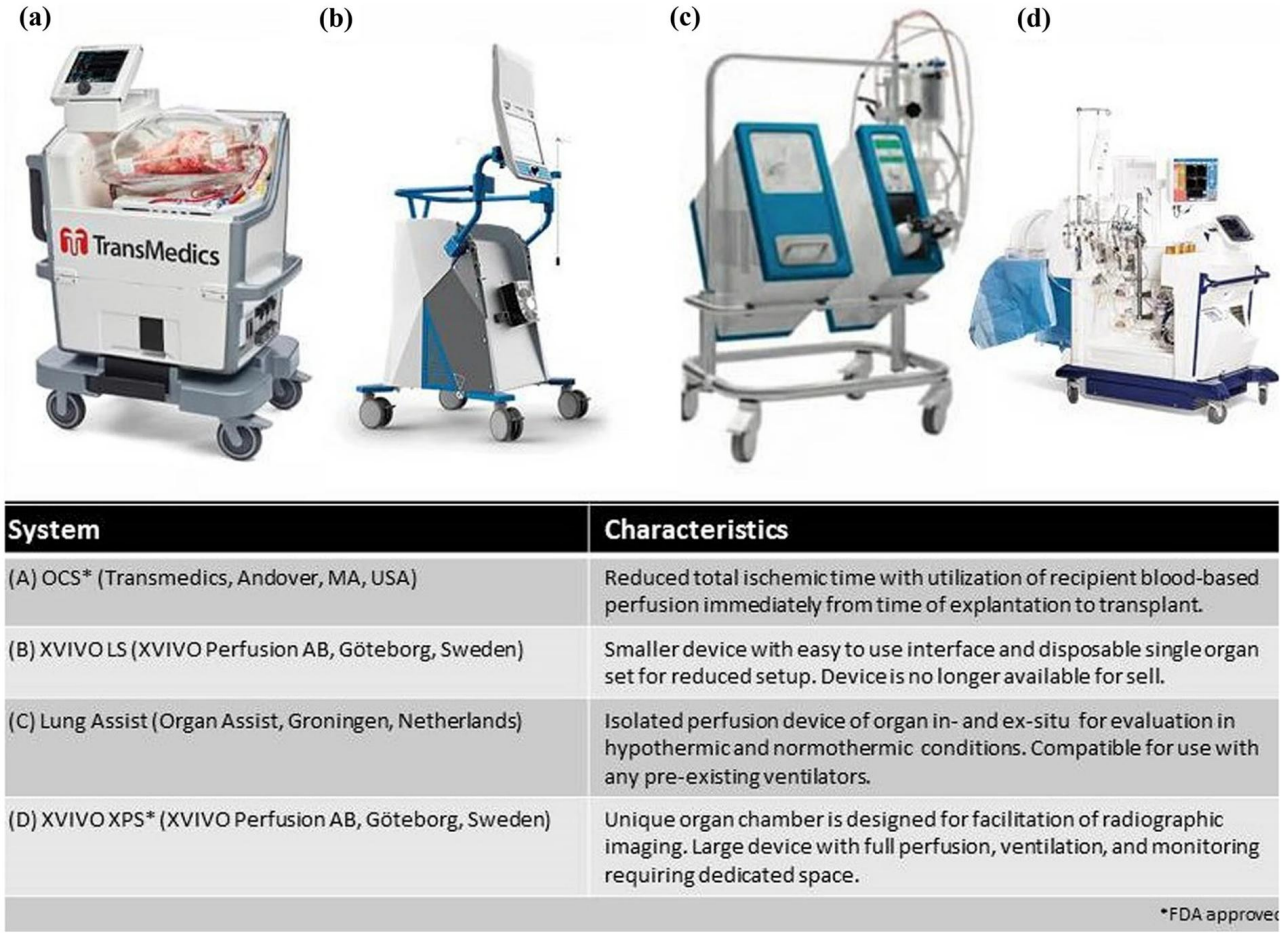
Commercially available EVLP systems. Reproduced from “Commercially available EVLP systems” by Kareem Ahmad, Jennifer L. Pluhacek and A. Whitney Brown, licensed under CC BY-NC 4.0.

## Normothermic regional perfusion

Once the determination is made to terminate life sustaining measures in DCD donors, comfort measures are instituted, and the patient is monitored for pulselessness. After an observatory period after pulselessness to check for signs of autoresuscitation, organ recovery begins. Prior to NRP, organ procurement was completed as quickly as possible to limit ischemic time and place the organs on perfusion devices. With thoracoabdominal NRP, the patient is cannulated via the ascending aorta and right atrium to reperfuse the donor organs prior to harvest, allowing the procurement teams to evaluate organ function prior to explant (2). This review is based on an expansive PubMed literature search for articles involving both normothermic regional perfusion and lung transplants. The initial search results identified 42 articles. After exclusion of editorials, studies involving only abdominal normothermic regional perfusion, and no inclusion of lung transplants in the results, nine studies remained. Cain et al. reported the first single-institution use of TA-NRP for DCD lung recovery. They analyzed a total of eight DCD donors who underwent TA-NRP with a lung protective protocol aimed at mitigating post-transplant pulmonary edema. Their strategy included aggressive donor diuresis, careful venous drainage at NRP initiation, and early pulmonary artery venting. The group ensured that all lungs were ventilated and reperfused *in situ* under controlled settings before procurement. They also performed functional assessment of the lungs which was based on airway inspection, recruitment response, oxygenation (P/F > 300 mmHg), and compliance. They found that TA-NRP, when combined with a targeted lung-protective approach, can expand the donor pool while maintaining outcomes comparable to conventional DBD lung transplantation (5). Chang et al. performed a single institution retrospective review from 2020 to 2023 comparing 11 lung transplants harvested from controlled DCD donors with TA-NRP and 138 lung transplants from donors after brain death (DBD). The primary outcome was primary graft dysfunction at 72 h and they concluded no statistically significant difference between the two groups, with a statistically significant decrease in ischemic time for the TA-NRP group (6). In concordance with Chang et al. findings, a retrospective review of all lung transplants in the United States from 2020 to 2022 found similar results. Of the 434 DCD transplants, 17 were recovered using TA-NRP. Compared to the direct recovery cohort, the TA-NRP population had a lower likelihood of mechanical ventilation >48 h and similar rates of predischarge acute rejection or requirement of extracorporeal membranous oxygenation (ECMO) (7). A similar study was done comparing lung transplants from 2019 to 2022 registered in the united network for organ sharing (UNOS) database. This study identified 627 DCD donors, of which 211 were procured using TA-NRP. Compared to the 416 donor lungs procured directly, the difference in lung utilization or 6 month post transplant survival was not statistically significant. Furthermore, the TA-NRP group had significantly lower rates of post transplant ECMO or mechanical ventilation (8). Another retrospective review confirmed the results from the above studies. They analyzed 987 lung transplants from 2020 to 2024 comparing direct procurement to TA-NRP and found no statistically significant difference between rates of PGD or 1-, 2-, 3-, or 12-month survival (9). A study from Spain evaluated 283 controlled DCDs from 2021 to 2023; 10% were harvested with TA-NRP and the other 90% using abdominal-NRP. The overall incidence of PGD was significantly lower in the TA-NRP cohort with overall survival being similar (10). The use of TA-NRP with regards to lung transplantation has also been described in the setting of cardiac donation including technical descriptions without lung allograft outcomes (11). Interestingly, a porcine study compared TA-NRP to direct procurement, both of which were followed by 3 h on EVLP. Various inflammatory cytokines and vital sign parameters were recorded throughout with histological evaluation of the donor lungs afterward. The authors found no difference in histology, cytokines, or metabolic profile of the TA-NRP lungs compared to DPP (12).

## *Ex-vivo* lung perfusion

First introduced in 2001, *Ex-vivo* lung perfusion (EVLP) has helped augment lung transplantation volumes (Figure 1) (13). It has expanded the donor pool by allowing for the evaluation and functional rehabilitation of donor lungs previously considered unsuitable for transplantation. Prior to EVLP, lungs were preserved using static cold storage, typically at 0°C–4°C (14). Although this method was effective at preserving procured lungs, it did not allow for assessment of the organ itself and significantly limited the number of organs that could be used (15). Additionally, prior to EVLP there was no reliable way to treat or recondition injured lungs before transplantation, which restricted the donor pool and contributed to high waitlist mortality. Static cold storage increases the risk of ischemia-reperfusion injury due to prolonged cold ischemic time. Without active perfusion and ventilation, metabolic waste accumulates and cellular injury progresses, which can impair graft function and increase the risk of primary graft dysfunction after transplantation (14). The Toronto Lung Transplant program was one of the first to describe the use of EVLP. In their pivotal trial, 23 lungs from high-risk donors received EVLP for 4 h, followed by transplantation. 116 lungs were stored via cool static storage. Outcomes were compared between the two groups of patients and patients who received EVLP treated lungs did not have an increased risk for primary graft dysfunction (PGD) or 30-day mortality. This was one of the first papers that established EVLP as a viable option for lung transplantation (16). As mentioned previously, EVLP allows for the assessment of procured lungs prior to implantation into a donor. A critical value that can be obtained with lungs and EVLP system is the P/F ratio. Arterial blood gas samples are obtained from the pulmonary vein while the lungs are ventilated with 100% FiO₂, and the resulting PaO₂ is used to calculate the P/F ratio. This provides an objective measure of gas exchange capacity in the perfused lungs (17). Pulmonary edema can also be assessed during EVLP. One method for assessing edema includes the “collapse” or “deflation” tests (18). This consists of disconnecting the ventilator at peak inspiration, and then visually assessing the lung to evaluate how effectively it deflates during passive exhalation. An x-ray of the lungs can also be obtained during EVLP, further enhancing the ability to detect pulmonary edema. Regarding timing, EVLP is most used for 3–6 h, which aligns with standard clinical protocols for evaluating and reconditioning donor lungs (19). However, with advances in perfusion technology and protocols, extended EVLP has been performed safely for up to 12 h in clinical practice. In experimental and investigational settings, durations beyond 12 h are being explored to allow for more prolonged assessment, therapeutic interventions, or logistical flexibility (20). In summary, EVLP is a technique that allows donor lungs to be perfused, ventilated, and evaluated outside the body under near-physiologic conditions. Clinically, it is used to assess and potentially rehabilitate marginal donor lungs, increasing the number of organs deemed suitable for transplantation, and has been shown to improve post-transplant outcomes. It is critical to denote that EVLP is a strategy used to preserve and rehabilitate lungs following procurement. This is in contrast to the TA-NRP which is used during the procurement process to optimize the lungs while *in situ*.

## Future directions

### Therapeutic

The benefits of EVLP in donor lung recovery, rehabilitation, and assessment have been discussed previously in this review. Another area of development for EVLP in lung transplantation is as a platform for delivery of therapeutic agents. This arena allows for organ specific drug activity without the common side effects with concerns for cardiotoxicity, hepatotoxicity, or nephrotoxicity. As advances are made in gene therapies these may be delivered directly to the donor lungs with the hope of benefits with regards to graft dysfunction and rejection (21).

### Barriers to widespread adoption

One of the primary barriers to widespread adoption of EVLP use, especially in low-income locations, is the upfront cost. It has been estimated to add an additional $50,000 on average to a lung transplant when compared to standard donors and cold storage preservation (22). Additional upfront cost may be expected in the training of staff from nursing, perfusionists, anesthesiologists, and surgeons for handling EVLP systems. This cost may be less as more volume is accumulated at a center utilizing EVLP and decreased post-transplant care costs, but the institutional costs of establishing a program's use of EVLP can be considerable. Further data on the real-world cost of EVLP use in lung transplant programs will be needed to convince lower resource groups and countries to adopt the technology.

### NRP protocols and long-term outcomes

The approach to NRP and EVLP in lung transplantation remains center specific. There are variations in the timing of NRP initiation, cannulation techniques, anticoagulation plans, ventilation strategies, and lung testing strategies. As more data is accumulated describing outcomes with each approach, it can be expected that best practices may be defined and outlined in expert consensus guidelines.

As a newer technology in the field of lung transplantation, long-term data regarding graft survival, chronic lung allograft dysfunction (CLAD), and patient quality of life (QOL) are limited. Development of prospective registry tracking specific to DCD recipients with EVLP usage is needed to assess clinical outcomes and identification of patient populations most likely to benefit from the usage (23).

It is critical to mention that technical standards for TA-NRP have been jointly endorsed by the Society of Thoracic Surgeons (STS), the International Society for Heart and Lung Transplantation (ISHLT), and the American Society of Transplant Surgeons (ASTS). These consensus guidelines describe procedural, ethical, and technical parameters for TA-NRP application across organ systems and include specific recommendations for lung procurement. These guidelines are aimed at standardizing practice and optimizing donor lung outcomes.

### Benefits to scheduling and standardization

Outside of the potential benefit to patient outcomes and expansion of the donor pool, it must be considered that EVLP may facilitate easier scheduling and less strain on the transplanting facilities and staff. As EVLP expands the safe procurement-to-transplant interval, the transplanting surgeon and staff may be able to operate during standard hours with the increases in institutional support that accompanies. This may provide a difficult to measure benefit to outcomes as surgeons and staff have more predictable working hours. The grafts will be thoroughly evaluated for function and patient matching. Patients may also have less onerous scheduling demands. These potential qualitative benefits to EVLP in lung transplantation will be difficult to measure but can be found in anecdotal reports (24).

## Conclusion

Lung transplantation is significantly limited by the available donor organs. Recent advancements in usage of DCD donors has led to an expansion of the available organs. Advances in organ evaluation, recovery, and rehabilitation have been facilitated with the use of normothermic regional perfusion in DCD procurement processes. Moreover, the development of *ex-vivo* lung perfusion (EVLP) has allowed for rehabilitation of donor lungs following procurement. These approaches become standardized and more-widely adopted. We can expect the donor pool for lung transplantation to continue to expand. Further advances may even lead to improved outcomes with organ-specific therapeutic options and advanced functional testing prior to transplantation.
